# Phase behavior and rheological properties of basil seed gum/whey protein isolate mixed dispersions and gels

**DOI:** 10.1002/fsn3.2148

**Published:** 2021-02-18

**Authors:** Vahideh Sarabi‐Aghdam, Seyed H. Hosseini‐Parvar, Ali Motamedzadegan, Saeed Mirarab Razi

**Affiliations:** ^1^ Department of Food Science and Technology Sari Agricultural Sciences and Natural Resources University Sari Iran; ^2^ Fonterra Research & Development Centre Palmerston North New Zealand

**Keywords:** biopolymers, dispersion and gel, phase behavior and rheology

## Abstract

Many food formulations comprise proteins and polysaccharides simultaneously, contributing in the functional properties in food systems. In this study, the effects of basil seed gum (BSG) addition to whey protein isolate (WPI) dispersions were investigated through phase behavior, steady shear flow, and small amplitude oscillatory shear tests (SAOS). The phase behavior of WPI‐BSG mixed solutions was dependent on the initial concentration of biopolymers, while the effect of BSG was predominant. Herschel–Bulkley model characterized the flow behavior of ternary mixtures, very well. Furthermore, apparent viscosity, the extent of thixotropy and viscoelastic behavior enhanced with increase in BSG concentration, significantly (*p* ˂ .05). Temperature sweep measurements showed a reduction in WPI gelling temperature by increase in BSG concentration. SEM results depending on BSG concentration revealed the protein continuous, bicontinuous, and polysaccharide continuous networks. Phase separation may be attributed to depletion flocculation and thermodynamic incompatibility of WPI and BSG molecules. The results confirmed the occurrence of phase separation and weak‐gel formation through mixtures, but the rate of gelation was more than the phase separation. In consequence, these results may open up new horizons in developing novel food products and delivery systems as well as utilizing as emulsifying, thickening and gelling agents in food and pharmaceutical industry.

## Graphical abstract

We studied the phase behavior and rheology of whey protein isolate (WPI) and basil seed gum (BSG) in the aqueous neutral pH medium. The results showed, WPI‐BSG mixtures had a Newtonian and shear‐thinning behavior depending on BSG concentration. The maximum strength of interactions in mixed gels obtained for the WPI‐BSG mixtures containing BSG >0.3%, wt. Phase separation was suppressed by formation of weak BSG gel in the mixture.

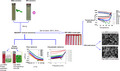

## 1 INTRODUCTION

Food products are a complex combination of different components, impeding the understanding of the role of each component in their interactions as well as the influence of different components on the characteristics of the final product (Picone & da Cunha, 2010). Proteins and polysaccharides are widely used to create the essential sensory features such as textural properties and control the release of flavor. Besides, they are efficient substances in controlling the colloidal stability of food systems (Goh et al., 2009).

Mucilage gums are natural adhesive polysaccharides, obtained from seeds or delicate stems of plants. All of mucilages are anionic polysaccharides with similar structures to some exudates gums. The increasing tendency to use them in specific food formulations is due to their great techno‐functional characteristics (vioscosifying, gelling, and thickening features) and their bioactive effects in treatment of specific diseases (Izydorczyk et al., 2005). Basil seeds provide adequate amount of mucilage gum (20% w/v). Rheological properties of basil seed gum (BSG) are comparable to xanthan gum, due to its high values of yield stress and strong shear‐thinning behavior (Hosseini‐Parvar et al., 2010). BSG is a promising hydrocolloid with interesting techno‐functional properties such as thickening, stabilizing, emulsifying, and texturizing, plus being fat substitute and surface‐active (Bahramparvar & Goff, 2013; Farahmandfar & Naji‐Tab asi, 2020; Farahmandfar et al., 2019; Naji‐Tab asi & Razavi, 2016, 2017a, 2017b; Razi et al., 2018, 2019, 2020). The carbohydrate content of BSG is about 79 (%wt). The crude BSG is a high molecular weight polysaccharides (2,320 kDa) consists of two factions including PER‐BSG (5,980 kDa, 69%) and SUPER‐BSG (1,045 kDa, 31%). It has a fibrillar structure containing scattered globules throughout the BSG polysaccharide backbone (Bahramparvar & Goff, 2013).

When aqueous mixtures of proteins and polysaccharides are prepared, four possible phenomena can occur, including cosolubility, thermodynamic incompatibility (segregative phase separation), depletion interaction (or flocculation), and complex coacervation (associative phase separation). Which phenomenon may occur in these mixtures depends on biopolymer concentrations and ratios as well as environmental parameters (pH, temperature, and salt) (Pérez et al., 2006; Yousefi & Jafari, 2019). When the macromolecules' affinity toward solvent is higher, segregative phase separation is promoted. On the other hand, when macromolecules carry net opposite charges, they form electrostatic complexes and aggregates, as a result of which associative phase separation occurs (Pérez et al., 2006; Schmitt & Turgeon, 2011; Xu et al., 2016; Yousefi & Jafari, 2019). In the conditions where one or both of the macromolecules tend to form gel network, the rate of phase separation process compared to gel formation determines the final status of the system. If gel formation happens, the phase separation will be inhibited (Ercelebi & Ibanoğlu, 2007; Lizarraga et al., 2006). Therefore, it is very important to study the phase behavior, gelling properties, and electrostatic interactions between these macromolecules, due to their effect on rheology and structure of food products (Neiser et al., 2000).

Whey protein isolates (WPI) and concentrates (WPC) are obtained from the whey, a byproduct of cheese manufacturing process. The major proteins in WPI with globular conformation and relatively low molecular weights are β‐Lactoglobulin (β‐Lg ~ 18 kDa) and α‐lactalbumin (α‐La ~ 14 kDa). Considering high proportions of β‐Lg and α‐La, the physicochemical properties of WPI system are related mostly on these proteins in comparision to other remained protein components as bovine serum albumin, immunoglobulins, and proteose‐peptones (Kontogiorgos et al., 2009). In addition to having beneficial nutritional characteristics, these proteins have considerable functional properties and are extensively utilized in production and stabilization of emulsions, gels, and foams in food systems, pharmaceutical industry, and cosmetics (Perez et al., 2009, 2010; Raoufi et al., 2017; Wagoner et al., 2016). Razi et al. (2019) found that foam density and foam stability of EWA significantly increased with an increase in BSG concentration (from 0% to 0.3% w/v). They also recently investigated the interaction of egg white albumin and BSG and found substantial and synergistic effect of BSG on egg white albumin (EWA) rheological behavior. It was reported that by increasing BSG concentration both flow behavior and viscoelastic behavior of mixtures were enhanced (Razi et al., 2020). In addition, we have previously applied BSG in sodium caseinate (NaCas) dispersions and gels in the presence of calcium chloride. It was successfully found that the microstructural, rheological, and phase behavior of systems were improved and the stability and water holding capacity of calcium sensitive NaCas dispersions and gels substantially enhanced (Sarabi‐Aghdam et al., 2020).

A better knowledge of the interactions between WPI and BSG at a neutral system (pH = 7) may help scientists to apply BSG in food systems, pharmaceutical industries and cosmetics. In this study, we aimed to explore the behavior and nature of interactions between BSG and WPI aqueous mixtures at neutral pH (away from isoelectric points of β‐Lg (~5.1) and α‐La (~4.3) and low ionic strength (NaCl, 0.05 M) (Kontogiorgos et al., 2009). Understanding these interactions would help someone to design complex food structures with specific properties and formulate new ingredients accurately for application in food systems as well as construction new delivery systems for functional foods, drugs and cosmetics. For this purpose, the current study was divided into two stages in order to obtain an excellent understanding of molecular interactions and forces; first, phase behavior of WPI‐BSG mixed systems was investigated along with steady shear flow behavior, and viscoelastic behavior, and then, the synergistic or antagonistic interaction between two biopolymers was studied by temperature sweep tests of WPI‐BSG mixtures and scanning electron microscopy.

## 2 MATERIALS AND METHODS

### 2.1 Materials

Basil seeds and whey protein isolate (WPI 895) were purchased from a local market (Mahhad, Iran) and Fonterra Co‐operative Group Limited, respectively. The chemical analysis of WPI based on the dry matter was 90.03% wt protein, 0.31% wt fat, <0.48% wt lactose monohydrate, 1.15% wt ash, and 4.45% wt moisture. All the chemicals used in this research were obtained from Merck.

### 2.2 Gum extraction

**FIGURE 1:**
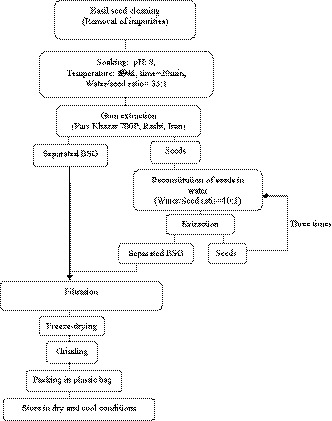
Extraction process of Basil seed gum

The extraction of BSG was performed according to method explained by Hosseini‐Parvar et al. (2010) (Figure 1).

### 2.3 Preparation of stock solutions and mixtures

The stock solutions of 1% w/v freeze‐dried BSG and 10% w/v WPI were prepared individually by gentle dispersing the powder into deionized water at ambient temperature and stirring for 1 hr at 1,500 and 500 rpm, respectively. Both stock solutions were finally overnight at 4°C to reach the maximum hydration. WPI‐BSG mixtures with different concentrations were obtained by mixing carefully weighed amounts of stock solutions and additional deionized water to prepare mixtures with certain concentrations of WPI (0.1, 0.3, 0.5, 0.7, 1, 2, 3, 4, and 5% w/v) and BSG (0.005, 0.01, 0.04, 0.05, 0.07, 0.1, and 0.3% w/v). The mixtures were stirred for 15 min until the components distribute homogenously throughout the system. The final pH value of the mixtures was adjusted to pH = 7.0 using NaOH (0.1–1 M).

### 2.4 Preparation of WPI/BSG mixed gels

Individual WPI and WPI‐BSG mixed gels prepared by protein concentration of 6% w/v as constant factor and BSG concentrations of 0, 0.05, 0.1, 0.3, and 0.5 (% w/v) as variable factor in ionic strength of 0.05 M NaCl and pH = 7.0. For this purpose, protein–polysaccharide mixtures were prepared in a 100 ml beaker with a diameter of 4.5 cm. 70 ml of the mixture was poured into the beaker and covered with aluminum foil and placed in a water‐bath with the temperature of 85°C for 30 min. After 30 min, beakers were exited and placed in a container of water and ice to reduce their temperature to about 10°C and then kept in the refrigerator, overnight (Beaulieu et al., 2001).

### 2.5 Phase diagrams

The WPI‐BSG solutions were stored in 10 ml tubes at 4°C. The phase behavior of WPI‐BSG systems was studied as follow: (a) *Aging for one day*. 24 hr after preparation of mixtures, they were centrifuged at 1,056.6 *g* for 15 min at ambient temperature to reveal the probability of phase separation. Phenol sulfuric acid and biuret assay were applied to determine the composition of upper and lower phases by measuring absorbance at 485 nm and 540 nm for polysaccharide and protein composition, respectively (Dubois et al., 1956; Layne, 1957). A series of different concentration of glucose anhydrous and BSA were prepared for construction the standard curves. (b) *Aging for one month*. The states of phases were visually investigated after one month of storage at 4°C. Phase diagram was established according to the initial concentrations of biopolymers. The volume of separated phases was reported as a percentage of the total volume. When the tubes containing ternary mixtures were inverted, depending on the visual observation of mixture flowing state, they were characterized as liquid when the mixture flows or gel when the material was gravitationally stable in the inverted tube. Moreover, they were more explored by small amplitude oscillatory measurements (Lazaridou & Biliaderis, 2009).

Phase separation in tubes of mixtures was visually controlled and the photographs of centrifuged samples were captured using a Cannon Powershot 2200, 14.1 Mega Pixel Camera. The visual phase diagram of samples was created using captured photos (Farouk et al., 2011).

### 2.6 Rheological measurements

#### 2.6.1 Flow behavior

The rheological measurements were performed with a Physica MCR 301 rheometer (Physica MCR 301, Anton Paar GmbH) using double‐gap geometry (properties). The sample was initially adjusted to rest for 5 min in order to reach the experiment temperature (20°C) (Hosseini‐Parvar et al., 2010). The Rheoplus software, version 3.4 (Anton Paar, Germany) was used for recording data. The shear rate was increased from 0.001 to 1,000 s^−1^ (upward curve) in the first cycle and then immediately decreasing to 0.001 s^−1^ (downward curve) in the next step. The downward flow curves were used for analyzing the flow behavior of the mixtures.

#### 2.6.2 Dynamic rheology

The linear viscoelastic (LVE) region was determined by an amplitude sweep test at strain range of 0.1‐100%, frequency of 1 Hz and 20°C. A frequency sweep test was performed for the mixtures at 20°C in the range of 0.01–10 Hz and strain of 3.5%. The viscoelastic behavior parameters including storage modulus (*G*′), loss modulus (*G*″), damping factor (tan *δ*), and complex viscosity (η*) were reported as a function of angular frequency (*ω*).The frequency dependency of *G*′ and *G*″ was determined using fitting these data by the power law model as follows:

(1) $$G^{\prime }=a\cdot \omega^{b}$$

(2) $$G^{\prime \prime }=c\cdot \omega^{d}$$

where, *ω* is angular frequency (rad/s), constants *of a* and *c* determine the magnitude of *G*′ and *G*″ at frequency of 1 Hz, respectively, while *b* and *d* shows the amount of frequency dependency for *G*′ and *G*″, respectively (Razi et al., 2020).

Temperature sweep tests were conducted for investigating the changes of *G*′ and *G*″ during heating and cooling process of WPI alone and in the presence of BSG were conducted. The temperature of samples equilibrated at 5°C. Then, the samples were heated from 5 to 95°C and then cooled from 95 to 5°C at the same rate of heating (at 2.5°C/min, 1 Hz and 3.5% strain). A thin layer of mineral oil was used to cover sample in order to prevent moisture loss during the measurement.

### 2.7 Microstructure

Gel specimens were prepared according to method of van den Berg et al. (2009) with some modifications. Samples (5 × 15 × 5 mm) were taken from the internal part of the gels with a razor blade and stabilized with 2.5% glutaraldehyde in 0.1 molar phosphate buffer for at least one night at 4°C. Glutaraldehyde acts as a stabilizing agent and provides cross‐linking in the protein. The stabilized specimens were then rinsed at least three times with 0.1 M phosphate buffer for 15 min. The washed gel samples were then dehydrated with increasing concentrations (30%, 50%, 70%, 80%, 90%, and 100%) of ethanol and then finally dehydrated with absolute ethanol. In order to complete the drying process, after washing with alcohol, the samples were placed in a freeze‐dryer for one day. Then, the specimens were broken up in a dry condition by a blade and placed on aluminum bases and covered with a thin coating of gold for six minutes. The microstructure of samples was analyzed by a scanning electron microscope and magnifications were ranged from ×1,000 to ×15,000 in broken sections.

### 2.8 Data analysis

The flow behavior data were fitted using several models including Power law, Casson, Herschel–Bulkley, and Bingham models. The determination coefficient (*R* ^2^) values were obtained to determine the best fitted model.

Herschel–Bulkley model was found the best fitted model with the experimental data among different flow models (data not shown), and it was used to describe the flow behavior of the mixtures:

(3) $$\sigma =k{\dot{\gamma }}^{n}+\sigma_{0}$$

where *σ* is shear stress (Pa), k is the consistency coefficient (Pa.s^n^), $\dot{\gamma }$ is the shear rate (s^−1^), n is the flow behavior index (dimensionless), and is the yield stress (Pa).

The area between upward and downward flow curves, that is, hysteresis loop area, was determined indicating the thixotropic behavior of the mixtures (Steffe, 1996). The following equation was used to calculate this area:

(4) $$\text{Hysteresis loop area}\;=\int_{{\dot{\gamma }}_{1}}^{{\dot{\gamma }}_{2}}k{\dot{\gamma }}^{n}‐\int_{{\dot{\gamma }}_{1}}^{{\dot{\gamma }}_{2}}k^{\prime }{\dot{\gamma }}^{n^{\prime }}$$

where ${\dot{\gamma }}_{1}$, ${\dot{\gamma }}_{2}$, k, $k^{\prime }$, and n, $n^{\prime }$ are initial shear rate,final shear rate, consistency coefficient, and flow behavior index for increasing and decreasing shear rate measurements, respectively (Razavi & Karazhiyan, 2009).

All measurements were carried out in duplicate in a completely randomized design. Minitab16 software (Minitab Inc., State College, Pennsylvania) was used to compare the mean value of different factors in Tukey's test (*p* < .05) and also to plot the graphs.

## 3 RESULTS AND DISCUSSIONS

### 3.1 Phase behavior of the mixtures

**FIGURE 2:**
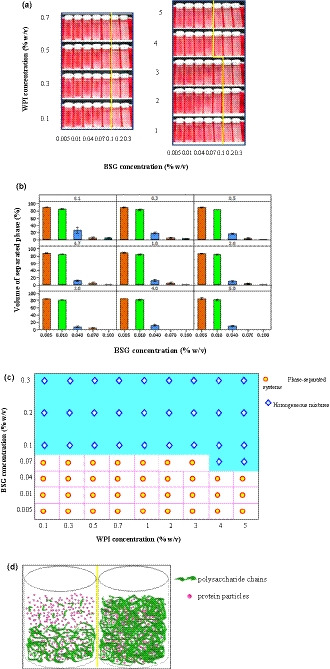
(a) Phase separation of WPI‐BSG mixtures after one day aging at 4°C and then centrifugation (1,500 rpm, 15 min); (b) Volume percentage of separated phases (translucent phase) resulting after one day aging at 4°C, representative aqueous dispersions of WPI‐BSG mixed systems.; (c) Bulk phase separation of WPI‐BSG aqueous dispersions after one month storage at 4°C; BSG: Basil Seed Gum; WPI: Whey Protein Isolate. d) Schematic representation of mixtures phase status

In general, a phase diagram depicts the behavior of systems containing two different polymers in a wide range of concentrations and ratios. Therefore, the complicated interactions between biopolymers in a mixture can be recognized by investigating the phase behavior (Jara et al., 2010). The visual appearance, phase diagram, and the volume proportions of upper transparent phase for different WPI‐BSG mixtures are presented in Figure 2. Immediately after mixing of BSG with WPI, a homogeneous dispersion was obtained. However, during the storage, the separation was occurred. The rate and amount of phase separation were dependent on BSG concentration. For most of aqueous mixtures, a very low concentration (0.1% w/v) of WPI was proper for ternary system to show immiscibility (Figure 2b & c). Phase separation occurred at very low concentration of BSG after storage and centrifugation (0.005% w/v), irrespective of WPI concentration. These results showed that while the upper phase was transparent, the lower phase was opaque, confirming the presence of associated polysaccharide chains.

At 0.1% w/v and higher concentrations of BSG, no macroscopically visual phase separation was observed and the phase separation was arrested and ternary mixtures were appeared to preserve their homogeneous liquid‐like appearance after storage for one day and also one month at 4°C. It is obvious that the volume proportions of upper and lower phase were dependent on the initial concentrations of both biopolymers, that is, when the initial concentration of BSG was low, the height of transparent phase was greater. The effect of WPI concentration at low concentrations of BSG was not clear; nonetheless, the effect of WPI concentration at concentration of more than 3% w/v was important and we observed both mono‐phasic and biphasic system in 0.1% w/v BSG. In the systems with phase separation and bottom opaque phase, as a result of moderately shaking by hand, the mixtures returned to their initial one‐phase appearance. Therefore, phase separation in ternary solutions was reversible. For WPI‐guar gum and WPI‐pectin phase‐separated systems, this phenomenon was reported to be attributed to relatively weak interactions between protein and polysaccharides (Ercelebi & Ibanoğlu, 2007). In lower concentrations of BSG, the amount of gum was not enough to distribute throughout the whole system, so phase separation was observed.

In the phase‐separated mixtures, the amount of protein and polysaccharide was compared with initial concentrations (Table 1). The analysis were just applied for mixtures containing 0.04 and 0.07% w/v BSG because the volume of upper and lower phase were not sufficient for conducting the measurement in other concentrations of BSG. Spectrophotometric results showed that protein concentrations in upper, lower, and initial mixtures were almost equal; however, the polysaccharide concentration was much higher in lower phase than upper phase (Table 1 and Figure 2d). Both BSG and whey proteins carry net negative charges at the neutral pH, so there is no interaction between BSG and WPI, making a relative stability due to electrostatic repulsion at the mixtures with low concentration of both biopolymers. However, self‐association properties of BSG molecules could change the stability of the mixture during the storage time (Lazaridou & Biliaderis, 2009). BSG has ability of weak‐gel forming and in contrast, WPI is a nongelling agent at lower concentrations, ambient temperatures, and neutral pH. When the entropy of a system increases and free energy of mixing approaches to the minimum, thermodynamic incompatibility of biopolymers occurs, resulting in the phase separation (Hemar et al., 2001).

**TABLE 1:** Protein and polysaccharide composition in initial WPI‐BSG mixtures and separated phases after phase separation (upper phase and lower phase)

Sample		Initial composition of ternary mixture		Upper‐phase composition		Lower‐phase composition	
WPI (%w/v)	BSG (%w/v)	Protein (mg/ml)	Polysaccharide (mg/ml)	Protein (mg/ml)	Polysaccharide (mg/ml)	Protein (mg/ml)	Polysaccharide (mg/ml)
0.7	0.04	7.341	0.583	6.659	0.148	7.886	0.986
0.7	0.07	7.386	0.907	6.159	0.298	7.591	0.989
1	0.04	9.727	0.52	9.318	0.143	9.726	0.916
1	0.07	9.864	0.896	9.045	0.295	10.93	0.973

These results were in agreement with phase separation of *iota‐*carrageenan and WPI mixtures (Ercelebi & Ibanoğlu, 2007; Lazaridou & Biliaderis, 2009). In *iota‐*carrageenan and WPI mixtures, the formation of homogenous gel prevented visual phase separation in all ternary mixtures (Ercelebi & Ibanoğlu, 2007).

### 3.2 Flow behavior of mixtures

**FIGURE 3:**
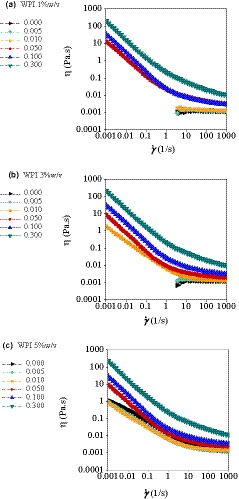
Typical flow curves of ternary (WPI‐BSG‐water) mixtures at different biopolymer concentrations (temperature: 20°C, NaCl: 50 mM): (a) WPI 1% different BSG concentrations, (b) WPI 3% different BSG concentrations, and (c) WPI 5% different BSG concentrations

The steady‐state flow behavior of WPI (1, 3 and 5% (w/v) of total mixture) and BSG (0.005%–0.3% (w/v)) mixtures were investigated. The rheological measurements were not applicable for the lower concentrations of WPI, due to the low viscous nature of the solutions. The flow curves of individual WPI and that mixed with BSG are shown in Figure 3. According to the obtained results, at low concentrations of WPI, the flow behavior of the mixtures was quite similar to the samples containing only WPI, showing Newtonian behavior with the same viscosity. (Figure 3a & b). However, when concentration of BSG was more than 0.05% w/v, a shear‐thinning behavior was more pronounced. According to Figure 3c, a non‐Newtonian behavior was observed for the WPI with concentration of 5% w/v, which could be attributed to the concentrated large particles and the WPI aggregates formed during the preparation (Hemar et al., 2001). The rheological behavior is expected to be governed by the rheology of continuous phase. The mixtures, used for the rheological measurements, were homogenous. Therefore, the continuous phase at low concentrations of BSG in the mixtures was WPI. In the biphasic systems, BSG increased the viscosity of the system and the apparent Newtonian behavior was disappeared, probably because the polysaccharide volume fraction was effective for the mixtures (Leng & Turgeon, 2007).

A change in continuity of the system was reported in β‐glucan and WPI systems (Kontogiorgos et al., 2009). At low shear rates, there is a strong shear‐thinning behavior with no Newtonian region, proving the existence of high zero shear viscosities at very low shear rates. This is the characteristics of polymer molecules with inflexible configuration, which have physical interactions with the neighboring polysaccharide chains (Hosseini‐Parvar et al., 2010). This behavior can be explained based on BSG intermolecular interactions forming aggregates, polymer entanglement, and weak gel formation surrounding the WPI solution in the network. When the shear rate increased, the particles were deformed and/or rearranged, resulting in lower flow resistance and consequently decreased viscosity, due to arrangement of molecules in flow direction (Hosseini‐parvar et al., 2010).

For the systems with non‐Newtonian behavior, several time‐independent models were fitted. For the flow behavior of the WPI‐BSG mixtures, the Herschel–Bulkley model was the best fitting model with higher *R* ^2^ values. The rheological parameters obtained using Herschel–Bulkley model from various mixtures are presented in Table 2.

**TABLE 2:** Rheological parameters from Herschel–Bulkley model for ternary mixtures at 20°C, 1 hr after preparation

WPI (% w/v)^†^	BSG (% w/v)^‡^	*σ*_0_ (Pa)	n(_)	*k* (mPa s^n^)	Hysteresis loop area (Pa s^−1^)	*R* ^2^
1	0.05	0.012 ± 0.001^e*^	0.834 ± 0.013^c^	9.354 ± 0.705^d^	30.590 ± 1.600^c^	.999
	0.1	0.022 ± 0.001^d^	0.908 ± 0.029^a^	5.872 ± 1.399^def^	29.050 ± 3.410^c^	.993
	0.3	0.143 ± 0.004^a^	0.705 ± 0.003^e^	74.197 ± 1.830^b^	126.600 ± 3.110^ab^	.999
3	0.05	0.002 ± 0.001^f^	0.834 ± 0.001^c^	3.135 ± 0.084^f^	7.370 ± 6.750^d^	.951
	0.1	0.023 ± 0.001^d^	0.903 ± 0.000^ab^	6.107 ± 0.018^def^	28.120 ± 13.188^cd^	.997
	0.3	0.164 ± 0.000^a^	0.739 ± 0.001^d^	59.618 ± 0.627^c^	115.600 ± 3.480^b^	.998
5	0.05	0.008 ± 0.001^e^	0.873 ± 0.003^b^	5.285 ± 0.627^ef^	6.035 ± 0.185^d^	.997
	0.1	0.019 ± 0.001^d^	0.884 ± 0.002^ab^	7.433 ± 0.056^de^	34.245 ± 11.255^c^	.998
	0.3	0.155 ± 0.000^a^	0.717 ± 0.003^de^	78.445 ± 2.697^a^	137.280 ± 3.430^a^	.999

^†^ Whey protein isolate.

^‡^ Basil seed gum.

* Values assigned with different letters are significantly different (*p* ˂ 0.05).

The consistency index (*k*) showed marked increase (*p* ˂ .05) for the mixtures of WPI (1%–5%, w/v) and BSG (0.05%–0.3%, w/v). Flow behavior index (n) was smaller than 0.9 in almost all of mixtures containing 0.05% w/v and higher BSG concentrations, and it decreased with the increase of gum concentration, implying the increase in shear‐thinning behavior and the increasing in shear stress with gum concentration. Farahmandfar et al. (2019) also reported the enhancement of shear‐thining behavior and decrease of flow behavior index at high concentrations of purified Basil seed gum solutions. Hosseini‐Parvar et al. (2010) attributed the decrease in the magnitude of n and the increase of k to the high molecular weight, size, and concentrations of biopolymers. By increasing the gum concentration, the yield stress value of the mixtures increased. When a system shows a high yield stress, it implies that it has high ability of holding and suspending the constituents homogenously in food system and can act as a stabilizer in food formulations. These results were in agreement with the findings of Razi et al. (2020) who worked on EWA and BSG mixtures. In addition, Leng and Turgeon (2007), who worked on WPI with κ‐carrageenan and pectin, and Sittikijyothin et al. (2010), who worked on β‐lactoglobuline and galactomannans obtained similar results. Sarabi‐Aghdam et al. (2020) investigated the flow properties of the NaCas and BSG mixed dispersions and the results were in line with these findings. Additionally, similar behavior was observed for λ‐carrageenan and WPC systems, too. Lizarraga et al. (2006) attributed this behavior to the limited thermodynamic compatibility of both biopolymers.

The time‐dependent rheological properties of WPI‐BSG mixtures are presented in Table 3 **.** Ascending and descending flow diagrams are representative of shear‐thinning (pseoudoplastic) behavior with a hysteresis area. The data of hysteresis loop area shows that by increasing BSG concentration, the extent of hysteresis loop area and as a result the thixotropy of system increase. Farahmandfar and Naji‐Tab asi (2020), Keisandokht, Haddad, Gariepy, & Orsat (2018) and Naji‐Tab asi and Razavi (2017b) previously reported thixotropic behavior for the BSG solutions. Farahmandfar et al. (2017) and Javidi et al. (2016) reported the increase of thixotropic behavior by addition of BSG in the whipped cream and ice cream, respectively. Higher concentrations of BSG form stronger connections between BSG polysaccharide chains. When a structure exists in dispersion and shear is applied on it, the structure collapses, causing the thixotropy (Farahmandfar et al., 2019; Koksoy & Kilic, 2004). BSG polysaccharide chains can make weak three‐dimensional network which is broken down under shear and shows thixotropy. It should be noted that when there is no shear, thixotropic materials are stable. In contrast, when the shear is applied, its viscosity decreases and easily pours from the containers such as mayonnaise and salad dressings (Farahmandfar & Naji‐Tab asi, 2020).

**TABLE 3:** Hysteresis loop area for WPI‐BSG‐water ternary mixtures

WPI^†^ (% w/v )	BSG^‡^ (% w/v)	Hysteresis loop area (Pa s^−1^)
1	0.05	30.590 ± 1.600^c^
1	0.1	29.050 ± 3.410^c^
1	0.3	126.600 ± 3.110^ab^
3	0.05	7.370 ± 6.750^d^
3	0.1	28.120 ± 13.188^cd^
3	0.3	115.600 ± 3.480^b^
5	0.05	6.035 ± 0.185^d^
5	0.1	34.245 ± 11.255^c^
5	0.3	137.280 ± 3.430^a^

^†^ Whey protein isolate.

^‡^ Basil seed gum.

* Values assigned with different letters are significantly different (*p* ˂ .05).

### 3.3 Frequency sweep test

**FIGURE 4:**
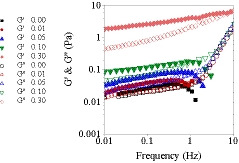
Dynamic frequency spectrum for mixtures of WPI 3% with different BSG concentrations

The effect of BSG concentration on storage (*G*′) and loss (*G*″) modulus of mixtures is presented in Figure 4. In the present research, frequency dependency of *G*′ and *G*″ moduli was shown only for systems containing 3% w/v WPI. The other concentrations of WPI (1 & 5% w/v) showed similar behavior (data not shown), due to the more significant and strong effect of BSG, governing the overall behavior of the mixtures. The results of frequency sweep measurements can be used for determination of the system behavior as dilute solutions, concentrated solutions and gels (Rao, 2007).

As can be seen in Figure 4, both moduli showed frequency dependency, that is, they increased with increasing frequency. The frequency sweep data extracted from Figure 4 are presented in Table 4. When the concentration of BSG increased from 0% to 0.3% w/v, *G*′ and *G*″ became much higher. In general, when *G*′ was much higher than *G*″, strength of structures was significantly higher. At low frequencies, it seemed that weakly flocculated particles of WPI and weak network of BSG in ternary mixtures contributed to the soft‐solid or viscoelastic‐solid behavior of the systems, in which there was only small stretch and relaxation between the existed bonds with very minor collapse and link together (Naji‐Tab asi & Razavi, 2017b).

**TABLE 4:** Rheological parameters of frequency sweep test at constant frequencies (1 and 10 Hz), cross‐over point, and the rheological parameters of WPI‐BSG dispersions after fitting storage and loss moduli data using Power law model

BSG concentration (% w/v)	10 HZ			1 HZ			Cross‐over point		*G*′ = *a*.*ω* ^b^			*G*″ = *c*.*ω* ^d^		
	Tan (*δ*) —	*G*″ (Pa)	*G*′ (Pa)	Tan (*δ*) —	*G*″ (Pa)	*G*′ (Pa)	*ω* (rad/s)	*G*′ = *G*″ (Pa)	*a*	*b*	*R* ^2^	*c*	*d*	*R* ^2^
0	∞	2.360	0.000	1.600	0.036	0.022	4.2	0.031	0.028	0.219	.909	0.022	0.203	.994
0.01	∞	1.900	0.000	0.998	0.038	0.038	5.05	0.036	0.039	0.197	.763	0.024	0.186	.975
0.05	∞	2.400	0.000	0.900	0.071	0.079	7.07	0.075	0.064	0.204	.900	0.040	0.258	.974
0.1	∞	2.290	0.000	0.913	0.161	0.176	7.23	0.172	0.131	0.162	.985	0.086	0.275	.954
0.3	1.080	6.880	6.360	0.532	2.140	4.030	52.7	6.240	2.893	0.183	.986	1.064	0.395	.963

At frequency of 1 Hz, *G*′ was 0.036 Pa. However, it increased to 0.161 and 2.140 Pa with addition of 0.1% BSG and 0.3% BSG, respectively (Table 4). The frequency dependency of *G*″ was more than *G*′ at higher frequency ranges, resulting in a cross‐over point (Table 4 and Figure 4), over which, the viscous behavior was dominant. Tan *δ* determines the ratio of *G*″ and *G*′ which are the two portions of viscoelastic behavior. For ideally elastic behavior, *δ* = 0 and there is no viscous portion (*G*″ = 0) and tan *δ* = 0. On the other hand, for ideally viscous behavior, *δ* = 90 and there is no elastic portion (*G*′ = 0) and therefore tan *δ* approaches infinity because of division by zero (Rao, 2007). Liquid‐like behavior was detected when *G*″ was much larger than *G*′, tan *δ* (*G*′/*G*″) ≫ 1 (Alijani et al., 2011; Rafe et al., 2012, 2013; Silva & Rao, 2007). The prompt increase of *G*″ might be due to the low molecular weight and hydrodynamic radius of some BSG molecules and WPI particles. Therefore, higher frequencies caused more relative movements and viscously dissipation of the deformation energy, in which there was not enough time to break bonds and entanglements to make new bonds before the next oscillating cycle. At neutral pH (=7), both WPI and BSG carried negative charges, being thermodynamically incompatible. Therefore, the repulsive forces and absence of strong attractive forces prevented the formation of strong structures, resulting in the production of a weak gel throughout these mixtures. Farahmandfar et al. (2017) also showed that the incorporation of BSG caused the formation of weak gel‐like network throughout the product. Similar curves were obtained by Lizarraga et al. (2006), who investigated the viscoelastic properties of whey protein concentrate and λ‐carrageenan aqueous mixtures.

**FIGURE 5:**
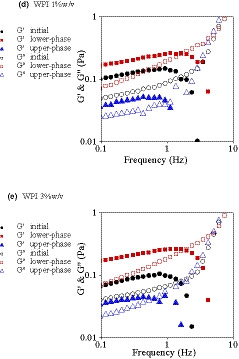
Dynamic frequency spectrum for phase‐separated systems containing 0.05% w/v BSG (initial mixtures, upper phase, and lower phase) after one day aging and 5 min centrifugation at 1056.6 g for clearly phase separation

The frequency dependency of *G*′ and *G*″ was more confirmed by the power law model. The obtained parameters (*a*, *b*, *c,* and *d*) are presented in Table 4. These parameters provide useful information on the viscoelastic nature of systems. When the magnitude of *b* and *d* were about zero, the *G*′ and *G*″ were not frequency dependent, a feature of elastic and covalent gel. In contrast, when *b* > 0, the frequency dependency increased and physical and/or weak gels was obtained (Hesarinejad et al., 2014; Mirarab Razi et al., 2018). In this research, the frequency dependency of *G*″ was more than *G*′, due to the higher magnitudes of *d* compared to *b*, confirming the cross‐over point occurrence observed in Figure 5. The magnitudes of *a* and *b* were in the range of 0.028–2.893 and *f* 0.219–0.162, respectively. By increasing BSG concentration, *a* increased, while *b* value decreased, demonstrating increased physical gel strength.

### 3.4 Verification of weak‐gel presence in mixed dispersions by Cox‐Merz rule

Complex viscosity, η*, indicates the viscous or elastic resistance of a structured sample to flow when oscillatory motions are applied on the sample. If the structured samples show higher resistance to flow, its complex viscosity will be greater and vice versa (Sharma, 2010). The Cox–Merz rule describes that in many polymer solutions, the magnitude of complex viscosity (η*) at a certain angular frequency (*ω*) in the small deformation (SAOS) measurements closely lay over to those of viscosity (*η_a_*) at a certain shear rate ($\dot{\gamma }$) in lage deformation (shear rate flow) measurements. This rule is only correct for isotropic solutions (Lizarraga et al., 2006). However, Cox‐Merz rule is not true for the systems whose components can aggregate and produce liquid crystals and consequently show weak‐gel properties with rigid polymer chains; therefore, they have values of η*(*ω*) > *η*(*γ*). Therefore, the Cox‐Merz rule has application in recognition of structured fluids from ordinary polymer solutions (Boyd, 2006).

**FIGURE 6:**
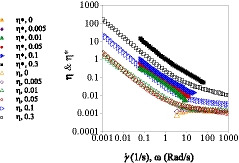
The effect of different BSG concentration on *Cox‐Merz* plot of mixture solutions of WPI and BSG

The downward flow diagrams from descending shear rate and the dynamic data from ascending oscillatory frequency were explored to demonstrate the correlation of Cox‐Merz rule. Figure 6 shows this rule correlation for WPI 3% w/v and different concentrations of the BSG mixtures. These systems did not obey the Cox‐Merz rule. At intermediate frequencies and shear rates, the η* placed over *η_a_*. When there was a hyper entanglement or aggregated structures in the system, the applied strain in oscillatory measurements was low, unable to destroy the existed and intermolecular associated structures. However, by applying higher shear rates in the large deformation test, the associated structures were disrupted, leading to higher η* in contrast to *η_a_*.

As Silva et al. (1993) suggested, departure from Cox‐Merz rule of waxy maize starch dispersions might imply a weak gel structure instead of a simple solution. Mirarab Razi et al. (2018) also reported deviation from Cox‐Merz rule for egg albumin‐BSG mixed gels. In addition, Farahmandfar and Naji‐Tab asi (2020) and Farahmandfar et al. (2019) showed the deviation from Cox‐Merz rules in systems containing different concentrations of BSG and purified BSG, respectively. Galactomanan samples also showed departure from Cox‐Merz rule, because of high density of entanglements and polymer–polymer interactions at low shear rates and frequencies. The departure from the Cox‐Merz rule was observed for High methoxyl (HM) dispersions, which were not true solutions. They were two‐phase systems with pectin micro‐aggregates dispersed into the solvent (Silva et al., 1993). The accuracy of Cox‐Merz rule was studied for 1% xanthan gum systems, showing that η*(*ω*) > *η*(*γ*), so the rule failed, due to the weak‐gel formation ability of xanthan gum (Boyd, 2006).

### 3.5 Temperature sweep test

**FIGURE 7:**
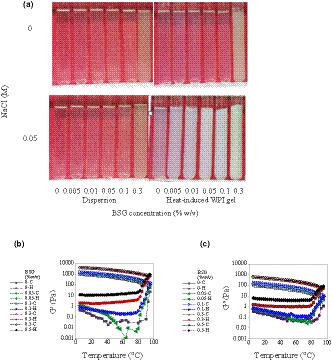
Effect of BSG concentration and ionic strength on gel formation of WPI (6% w/v)( a); Variation of storage modulus (*G*′) for WPI dispersions (6% (w/v) protein, neutral pH, ionic strength 0.05 M) at different BSG concentrations as a function of temperature (1 Hz, 2.5°C/min and 3.5% strain); (b) Heating from 5 to 95°C and (c) cooling from 95 to 5°C

The preliminary experiments for the gelation of WPI by heating the WPI dispersions at 85°C for 30 min in a glass tube showed that transparent heat‐set gels of individual WPI were obtained at 5% w/v of biopolymer in the presence of salt (0.05 M NaCl, neutral pH). Figure 7a shows the visual observation of individual and mixed WPI gels. When a salt was added to the protein solution, the negative charges of the protein disappeared and repulsion between particles reduced, resulting in the self‐association of protein particles and formation of self‐supporting gel (Totosaus et al., 2002). In addition, at the neutral pH (i.e., pH = 7), the electrostatic repulsive forces prevented the aggregation of WPI molecules, ending in the formation of a transparent/translucent fine‐stranded gel structure.

Temperature sweep tests were run to study the gel‐forming mechanisms of 6% w/v WPI alone and mixed with different concentrations of BSG. When protein concentration was above the gel critical concentration and heating was applied to the protein solution (usually above 60°C), protein particles denatured and aggregated, resulting in the formation of gel (Li et al., 2006). Figure 7b shows the effect of heating on storage modulus of individual WPI and that mixed with BSG dispersions. The gelling temperature (T_gel_) was determined by plotting *G*′ versus temperature in a linear scale and the sharp increase in *G*′ was regarded as T_gel_. It can be found that addition of BSG decreased T_gel_ of WPI solutions, and gelation occurred at lower temperatures by heating from 5°C to 95°C. After the sharp increase in the storage modulus, it increased uniformly during heating to 95°C. Hosseini‐Parvar et al. (2010) showed the increase of *G*′ of 1% BSG, while the temperature increased from 40 to 85°C, attributable to the strengthening of hydrophobic interactions during heating at above 60°C. Such a decrease in T_gel_ had been previously observed for WPI‐xanthan gels (Bertrand & Turgeon, 2007) and β‐lactoglobulin/BSG gels (Rafe et al., 2013). The increasing trend depended on BSG concentration, that is, at higher BSG concentrations, the slope of *G*′ versus temperature was more than that at lower concentrations and the solution was thickened more at the higher concentrations (Rafe et al., 2013). The presence of BSG in the mixtures increased the local concentration of WPI and promoted the aggregation and gelation process of WPI and segregative phase separation of mixtures, resulting in the highest *G*′ for 0.3% and 0.5% (w/v) BSG. The visual observation of the gels became turbid, probably as a consequence of BSG‐rich inclusions. Sanchez et al. (1997) and Bertrand and Turgeon (2007) reported the similar results for their mixed gels of xanthan‐WPI and xanthan‐WPI gels, respectively. Phase separation results in the increase of protein concentration in protein‐rich phase and promotes the aggregation rate (Li et al., 2006).

The storage modulus of the samples increased during cooling from 95°C to 5°C (Figure 7c). A small but noticeable decrease in *G*′ for 0.3 and 0.5% w/v BSG was observed at the initial times of cooling, and then it was increased gradually. This decrease in *G*′ might be due to reduction in hydrophobic interactions between BSG molecules, strengthening at higher temperatures (Rafe et al., 2012, 2013). Such a decrease was reported for individual BSG solutions (Rafe et al., 2012, 2013). These results revealed that BSG became the continuous phase and its characteristics governed the systems. For lower concentrations of BSG, this reduction in *G*′ was not observed. By cooling from 95 to 5°C, the storage modulus increased gradually, suggesting the continuity of gel formation process due to formation of new hydrogen bonds through BSG molecules (Rafe et al., 2013). These results were in good agreement with those obtained by Rafe et al. (2012), who studied the gelation of β‐lactoglubolin (10% w/v)/ BSG (1% w/v) mixtures. This observation might be due to a reduction in entropy, reinforcing the attractive forces (hydrogen bonding, van der Waals forces) between the protein particles, and presence of covalence and hydrophobic interactions between molecules, providing firmer gel structure (Rafe et al., 2013).

### 3.6 Morphology of WPI‐ BSG mixed gels

**FIGURE 8:**
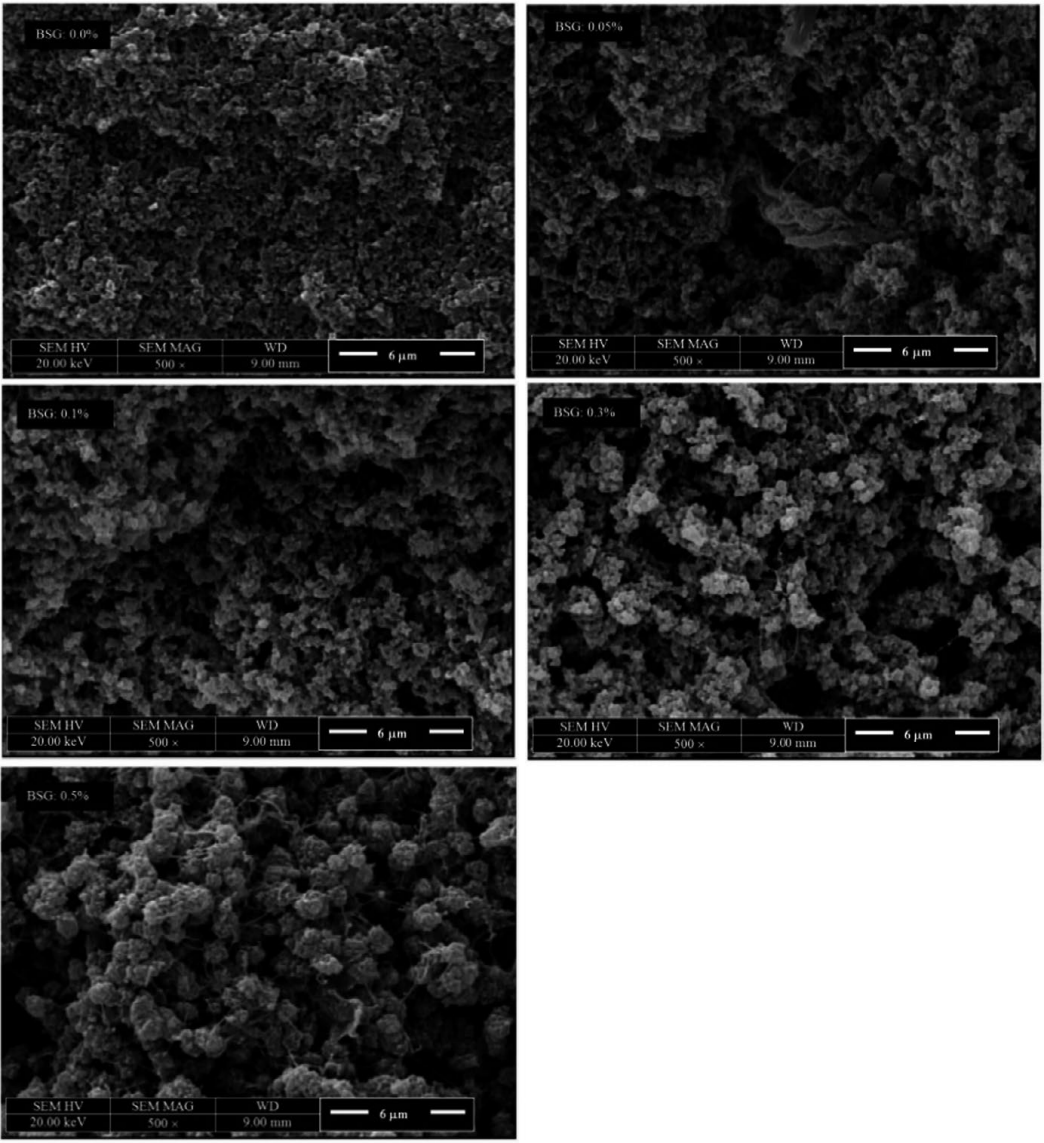
Effect of BSG concentration 0, 0.05, 0.1, 0.3, and 0.5 (% w/v) on heat‐induced WPI gels microstructure at 5,000× magnification

**FIGURE 9:**
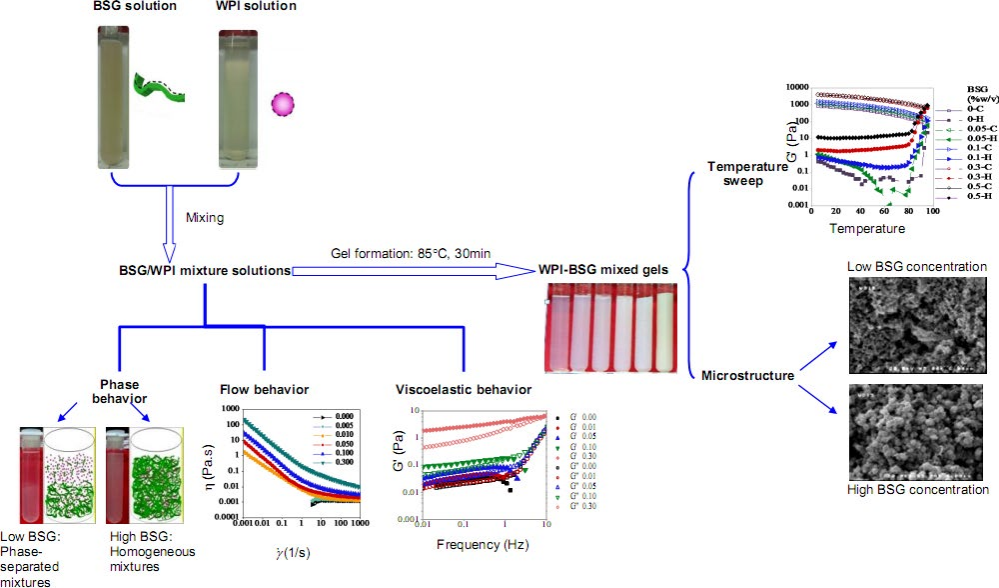
Graphical abstract

Scanning electron microscope (SEM) provides some information about the microstructure of biopolymers containing proteins, polysaccharides and their mixed systems (Rafe et al., 2013). Microscopic images of WPI‐BSG mixed gels, as a function of BSG concentration, are presented in Figure 8. At low BSG concentrations (i.e., 0.05 and 0.1% w/v), WPI formed large globular aggregates, connecting together and maintaining water in structure by producing compact gel. The polysaccharide‐rich phase, compared to the dispersed phase, was distributed throughout the WPI gel network, confirming the protein continuous phase (Figure 8). Average particle size of WPI molecules were about 1 μm and some small void spaces were observed. By increasing the BSG concentration to 0.3% w/v, the continuous phase changed to bicontinuous (Figure 8). In addition, the size of pores in the structure of 0.3% w/v BSG were larger, disrupting the protein network and the aggregated proteins were more elongated. The addition of 0.5% w/v of BSG resulted in the aggregation of small protein compared to 0.3% w/v of BSG. These aggregated proteins were homogenously distributed throughout the BSG continuous phase and formed the finer network structure. WPI‐BSG interaction led to new network structure with enough water holding capacity and desired properties. In a system in which two negatively charged biopolymers (e.g., proteins and polysaccharides) were present and they were thermodynamically incompatible, the ultimate behavior of the systems depended on the rate of phase separation or gel formation. The gels formed in the neutral pH were phase‐separated. This type of structure was reported in another work, where k‐carrageenan formed the continuous phase with milk protein aggregates dispersed phase (Lazaridou & Biliaderis, 2009). The formation of β‐lactoglobulin continuous gel with high water holding capacity was reported in previous studies (Hosseini‐Parvar et al., 2015; Rafe et al., 2013). In addition, they reported the biphasic gel network for β‐lactoglobulin and BSG mixed systems containing large pores at higher BSG concentrations, attributed to high concentration of β‐lactoglobulin, as the main heat‐set gelling component of whey proteins (Figures 8 and 9).

## 4 CONCLUSION

In this study, the phase behavior and rheology of basil seed gum/whey protein isolate mixtures and gels were investigated. The difference between the rates of phase separation and gelation, and the fact that which component is the continuous phase governs the system behavior. The viscosity, pseudoplasticity, and yield stress of the mixtures and hysteresis loop area increased with an increase in the concentration of BSG. Furthermore, frequency sweep data, similar to flow behavior results, confirmed the presence of weak gel in the systems where spectra moduli increased with the increase of the BSG concentration. Based on the results of studying the gel formation process, the WPI gel structure was strengthened, due to thermodynamic incompatibility and segregative phase separation. The microstructure of ternary dispersions and gels proved the presence of WPI, bi‐, and BSG continuous networks, as confirmed by the rheological measurements and SEM micrographs for the mixed gels. The rheological measurements revealed that BSG concentration had synergistic effect on the formation of WPI gels with new structures at the neutral pH. As a consequence, the incorporation of BSG into WPI gels might improve the textural properties, due to which even at 0.05% w/v of BSG it had significant effect on the obtained gels. The results of this study provides useful insights for quality controlling of food products and creating new food structures, utilizing as thickener, stabilizer, and encapsulating bioactive materials with controlled release goals.

## CONFLICT OF INTEREST

The authors declare that there is no conflict of interest.

## ETHICAL APPROVAL

This research does not include any human or creature testing.

## Data Availability

The data will be available from the authors upon request.
